# Case report: Post-traumatic Tourette syndrome resolving with treatment of jugular venous narrowing; reconciling organic brain dysfunction following whiplash trauma with the absence of direct brain injury

**DOI:** 10.3389/fneur.2023.1179596

**Published:** 2023-06-05

**Authors:** J. Nicholas P. Higgins, Stephen Kirker

**Affiliations:** ^1^Department of Radiology, Addenbrooke’s Hospital, Cambridge, United Kingdom; ^2^Department of Rehabilitation Medicine, Addenbrooke’s Hospital, Cambridge, United Kingdom

**Keywords:** Tourette syndrome, whiplash injury, cerebrospinal fluid leak, jugular vein stenosis, functional neurological disorder

## Abstract

We describe a man aged 33 years who developed multiple symptoms, personality change, and a severe tic disorder following a road traffic accident, which were undiminished for 3 years until jugular venous narrowing between the styloid process of the skull and the transverse process of the C1 vertebra was treated by surgical decompression. Immediately following surgery, his abnormal movements almost completely resolved, with no regression in 5 years of follow-up. Vigorously debated at the time was whether or not his condition represented a functional disorder. Unrecognized throughout his illness, however, was a complaint of intermittent, profuse discharge of clear fluid from his nose that began on the day of the accident and continued up to the time of surgery, after which it was substantially reduced. This outcome reinforces the idea that jugular venous narrowing can cause or perpetuate a cerebrospinal fluid leak. It suggests that the interaction between these two pathological defects may have a profound effect on brain function in the absence of any demonstrable brain lesion. It invites a reevaluation of normal head and neck venous anatomy. It should strike a cautionary note in the diagnosis of functional illness. It invites exploration of a remediable structural cause for Tourette syndrome.

## Introduction

Tourette syndrome refers to a condition of involuntary motor and vocal tics of unknown cause, arising spontaneously in childhood and sometimes lasting a lifetime (1). Onset can be insidious or dramatic. Tics may be minor and easily disguised or severe and disabling (2). Not strictly Tourette’s are cases, otherwise identical, in which there seems to have been a precipitating event even though the underlying pathophysiology is unknown, traumatic brain injury being one. Yet the relationship between traumatic brain injury and tics is unclear (3); cases have been described following whiplash neck trauma or peripheral injury in which brain injury seems to have been minimal or absent (4, 5).

We describe a patient who developed a severe movement disorder, indistinguishable from Tourette syndrome, in the days after a road traffic accident in which he sustained a thoracic fracture but no evidence of brain injury. While he was under medical care, there was considerable debate as to whether his symptoms represented a functional disorder or were the result of an organic disturbance of intracranial pressure. However, 3 years after the onset of symptoms, partial relief of jugular venous narrowing brought an immediate and almost complete resolution of abnormal movements, with no regression in 5 years of follow-up.

## Case report

### History and initial investigations

A man aged 33 years was taken to the hospital after colliding with a car while riding his moped. There had been no loss of consciousness. There were no abnormal neurological findings. A CT brain scan was normal, and he was discharged after 6 h. The same night, he became confused, unable to see clearly, unsteady, and vomiting. He was brought back to the hospital but discharged again after a few hours. He was admitted 4 days later with increasingly aggressive behavior and numbness and weakness down his left side. A T9 compression fracture was treated with a brace. In the hospital, 7 days following the accident, he began to develop tics, first as minor oral movements, progressing over a matter of days to substantial vocal and motor spasms, the latter affecting his head, neck, trunk, and upper limbs. He also complained of headaches, visual disturbances, slurred speech, word-finding difficulties, and short-term memory impairment. Brain MRI was normal, and he was discharged.

When examined at our institution 2 months later, he was virtually housebound with a florid syndrome of tics and involuntary vocalizations. Abnormal movements included a stammer, humming, facial grimacing, and shoulder shrugging. Vocalizations involved clang associations “tick tock, rock rock” and repeated profanities. All were suppressible for short periods at the expense of extreme discomfort and emotional upset. All were exacerbated by anxiety and partially relieved by distraction. He was walking on crutches. MRIs of the brain and spine were unremarkable. There was no family history of Tourette syndrome.

Over the next 3 years, his tics were undiminished. There were no objective neurological findings, and there was broad agreement between neurology and psychiatry that his symptoms were largely functional. He was reviewed by the ophthalmology service, who recorded grossly disordered eye movements and grossly restricted visual fields but no papilledema. His premorbid personality had been boisterous and outgoing. After the accident, his mood varied between elation and depression, with outbursts of anger. With time, he became apathetic, fatigued, and withdrawn. His weight increased, and he noticed reduced facial hair and libido. Pituitary function tests were all normal except that gonadotrophin levels were not increased in the face of low testosterone levels. He was started on testosterone replacement therapy. He developed polydipsia, which was attributed after investigation to a constant sensation of dryness in his mouth.

### Investigation of intracranial pressure and cranial venous outflow

**Figure 1:**
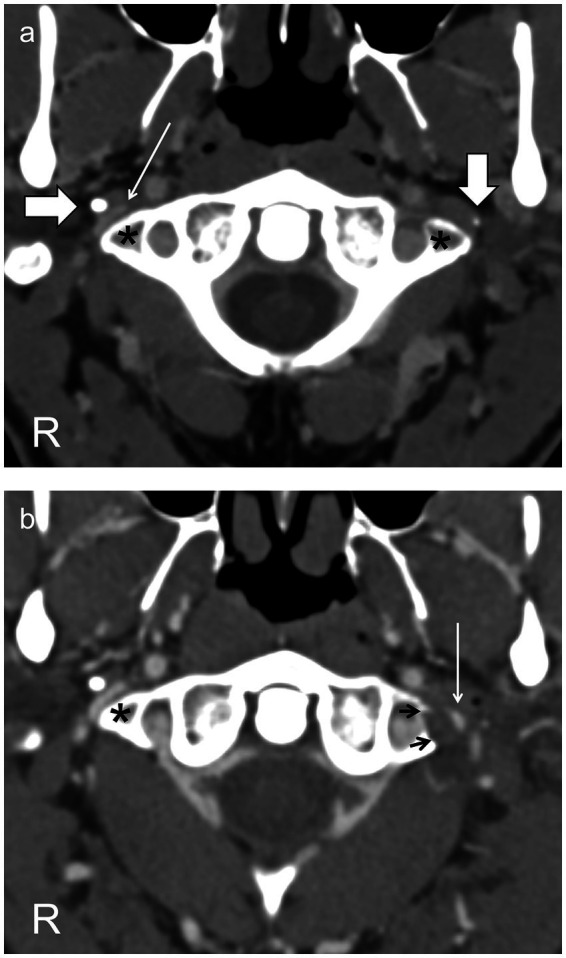
Axial CT scans through the C1 vertebra following intravenous contrast **(A)** before surgery show the jugular vein on the right side (thin arrow) markedly narrowed between the styloid process (horizontal thick arrow) and the C1 transverse process (asterisk) and on the left side compressed into invisibility between the styloid process (vertical thick arrow) and the C1 transverse process (asterisk). **(B)** After resection of the left styloid and C1 transverse process (resection margin, black arrows), the left jugular vein (thin arrow), though still narrowed, is now visible. The right jugular vein is unchanged, as expected.

**Figure 2:**
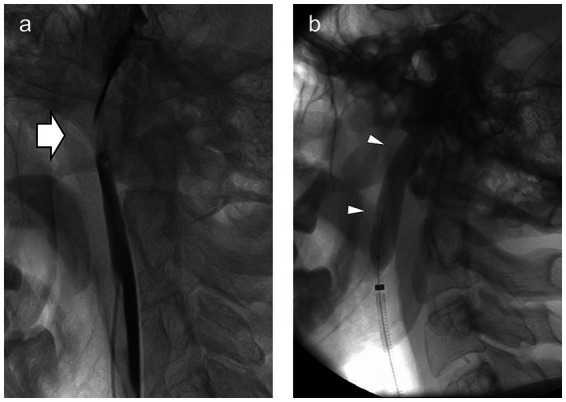
**(A)** Lateral view of the left jugular venogram showing marked narrowing of the jugular vein (arrow) between the (unseen) C1 transverse process and the (unseen) styloid. Intravascular contrast is dark, outlining the jugular vein above and below. **(B)** Same view with the angioplasty balloon (arrowheads) inflated across the site of narrowing.

With intractable symptoms and our group’s interest in disordered cerebrospinal fluid (CSF) dynamics and cerebral venous outflow obstruction, he underwent CT venography. This showed normal intracranial venous sinuses but marked narrowing of both jugular veins between the styloid processes of the skull and the transverse processes of the C1 vertebra (Figure 1A). Lumbar puncture revealed an opening pressure of 20 cm H_2_O, and his headache responded temporarily to cerebrospinal fluid (CSF) drainage, although tics were unchanged. Catheter venography confirmed the jugular narrowings, each associated with a 3 cm H_2_O gradient (Figure 2A). He had bilateral jugular venoplasty (Figure 2B) with no immediate effect, but over the following week, his physical and vocal tics were greatly reduced. His headache had improved. His demeanor was calmer. His head felt clearer, and his memory was improved. Within 2 weeks, all symptoms had returned (see Supplementary Videos S1, S2).

### Treatment of obstructed cranial venous outflow

He was diagnosed with cranial venous outflow insufficiency and, following appropriate counseling, had resections of the left styloid process and of the left C1 transverse process in a single procedure (Figure 1B) (6, 7).

The next day, barring the occasional facial tic, all his abnormal vocalizations and abnormal movements had resolved. His headaches, balance, and mood improved. At ophthalmology review 6 weeks later, his vision had returned to normal.

### Progress

His headache reoccurred in the months following surgery, and there was residual mood and cognitive disturbance, as well as profound fatigue. He responded to repeat venoplasty, but further surgical and stenting procedures were not successful in effecting any further reduction in jugular narrowing or seemingly any lasting clinical benefit.

A review of his notes 2 years after his initial intervention revealed a brief mention of a fluid discharge from his nose around the time of the accident. This was not addressed by his treating physician at the time, but now, on direct questioning, his partner recalled copious volumes of clear fluid coming from his nose on the night of the accident, adding to the distress experienced by the family on his arrival home. Then, over the next 3 years, this nasal discharge was repeated frequently, with no obvious precipitating factor, sometimes, for example, when they were sitting together watching television. However, none of this was revealed in numerous follow-up medical consultations, and at no time prior to surgery was the possibility of a CSF leak considered.

Following surgery, these discharges were substantially reduced in frequency and volume, and late attempts to establish the presence or likely origin of a CSF leak were unsuccessful. Then, at around 3 years post-surgery, they ceased altogether, this coinciding with a pronounced improvement in his general health and restoration of his premorbid affect, although headache and fatigue have persisted, if less severe than previously (see Supplementary Video S3).

## Discussion

There were two differing interpretations of this case while he was under medical care: the first was that a road traffic accident had precipitated a functional neurological disorder, and the second was that a road traffic accident had precipitated an organic disturbance of brain function from obstruction to cranial venous outflow caused by traumatic damage to the jugular veins. A third interpretation, possible only in retrospect, is that the accident caused a dural tear and CSF leak, which only healed after a procedure that improved cranial venous drainage, and that symptoms were largely a manifestation of CSF depletion syndrome (8–11), though unusual in the severity of the movement disorder and the absence of a postural component to headache (12).

The development of tics, or recrudescence of a tic disorder, after head injury is well recognized (3). Sometimes this can be attributed to damage to particular brain structures, but when there is no radiological evidence of injury, this attribution becomes more speculative (4, 5). Moreover, new-onset tics have been reported after whiplash trauma when there has been no loss of consciousness and no apparent brain injury (4, 5). In these circumstances, psychological mechanisms are likely to be invoked to explain the clinical picture, and when, as in the case we describe, there is such a multiplicity of symptoms and inconsistent neurological signs, it is inevitable that the differential diagnosis will include a primary psychiatric illness or functional neurological disorder.

What then prompted investigation of intracranial pressure and cranial venous outflow? CT venography showed narrowing of the jugular veins between the styloid processes of the skull and the transverse processes of C1. However, there were no signs of raised intracranial pressure. Moreover, there is no recorded association of venous obstruction or raised intracranial pressure with Tourette syndrome, and his other symptoms—headache, visual disturbance, nausea, vomiting, gait ataxia, fatigue, mood disturbance, cognitive disturbance, and memory disturbance—though frequent when intracranial pressure is chronically raised (as in idiopathic intracranial hypertension, IIH) would generally be regarded as being non-specific, not least because many of the same symptoms are also seen when intracranial pressure is chronically depressed (as in spontaneous intracranial hypotension) (8, 12–15).

Yet these two conditions can be connected. Thus, IIH is a disorder of raised intracranial pressure of unknown cause, arising spontaneously, mainly in obese young women (13). Spontaneous intracranial hypotension refers to a condition of low intracranial pressure caused by the spontaneous development of a CSF leak (8). Headache and visual disturbance are the signature complaints of the first, and postural headache of the second. Increasingly, however, spontaneous intracranial hypotension is being seen as complication of IIH, developing when the dural lining of the subarachnoid space, attenuated by chronically elevated intracranial pressure, gives way at a weak point (16–18). Intracranial pressure may be in the normal range in these circumstances (8, 16), and the characteristic features of either condition may be absent, leaving the multiple other symptoms that are found in both (8, 14, 15). So, are these other symptoms simply the psychological accompaniments of chronic illness, or are they evidence of a disorder of intracranial pressure? (19).

A recent study suggests the latter. Two studies, one exploring chronic fatigue and the other fibromyalgia, have found mean intracranial pressures to be in the high normal range and that patients are symptomatically improved by CSF drainage. Both conditions are characterized by the multiple symptoms cited above, and the clinical improvement seen with lumbar puncture applied not just to headache (usually present) but to many of the other symptoms as well (20, 21). Various combinations of headache and these other complaints, therefore, rather than confounding the classical features of disordered intracranial pressure, may, in fact, be relatively strong indicators of a pressure disturbance (22).

In the case we describe, observing bilateral jugular venous narrowing and using these symptoms as a signal to investigate intracranial pressure further led to a diagnosis of cranial venous outflow obstruction, in retrospect accompanied by a CSF leak. This led, in turn, to a procedure designed to improve cranial venous outflow by creating space for the left jugular vein to expand, and the result was an immediate and almost complete cessation of abnormal movements along with (also in retrospect) a reduction in overt signs of CSF leakage. This lends support to the original diagnosis, and although a placebo effect cannot be excluded, his nuanced response to surgical intervention with respect to his CSF leak and other symptoms, in keeping with the gradual healing of a dural defect and reflecting the limited extent to which the intervention was successful in relieving venous obstruction (Figure 1B), suggests otherwise.

There are precedents for this approach in the literature. The development of pseudomeningoceles or CSF leaks following vestibular schwannoma resection, for example, has been linked to iatrogenic occlusion of the sigmoid sinus during surgery (23, 24). In these cases, a chronic mild elevation of intracranial pressure, caused by obstruction to cranial venous outflow, is hypothesized to maintain a pressure gradient across the surgical defect in the dura, preventing closure. Treatment by revascularization and stenting of the sinus removes the force driving the elevation of intracranial pressure and allows the dural defect to heal.

Similarly, addressing jugular venous narrowings in cases of spontaneous intracranial hypotension by removing the driving force tending to elevate intracranial pressure in the first place can allow a dural defect to heal on its own (25, 26). Thus, cranial venous outflow obstruction might cause a CSF leak or may perpetuate a leak if a leak has developed for another reason. Moreover, symptoms may be complex, reflecting the balance between the clinical effects of the primary pathology (venous obstruction), the mitigating influence of a CSF leak on intracranial pressure, and the compounding problem of CSF depletion on brain function (22).

In practice, attributing clinical significance to jugular venous narrowing is difficult. The styloid processes are often quite closely approximated to the transverse processes of C1, and jugular venous narrowing at this site is seen frequently enough in radiological practice that it does not usually invite comment (27). Moreover, the pressure gradients associated with skull base or extracranial venous obstruction are usually not impressive (24–26, 28–30). Yet, this anatomical configuration would seem likely to place the jugular veins at risk of damage in a whiplash injury (in fact, the small size of the jugular veins at this level in this case might reflect scarring from previous trauma), and the outcome here suggests that in the appropriate context, these radiological findings should be taken seriously.

Determining which symptoms are due to a CSF leak and which are due to cranial venous outflow compromise is also difficult. The explanation for movement disorders in spontaneous intracranial hypotension is speculated to be in the distortion of brain structures, and in the stretching of cranial nerves, which occurs when CSF is depleted (10, 19). No brain distortion was seen here. Thus, it is questionable whether a CSF leak would be necessary to cause the clinical syndrome we observed. Nevertheless, this case suggests a substrate for post-traumatic movement disorders that would link them with acquired CSF leaks, these leaks caused, exacerbated, or prolonged as a result of damage to the jugular veins from a whiplash neck injury. This etiopathological mechanism is easily reconciled not only with their association with traumatic brain injury but equally with its absence. Moreover, if this is the template for post-traumatic movement disorders, it will likely have relevance for other more subtle cognitive and psychological disturbances occurring in the same circumstances (31). It may also be relevant to non-traumatic Tourette syndrome.

## Data availability statement

The original contributions presented in the study are included in the article/Supplementary material, further inquiries can be directed to the corresponding author.

## Ethics statement

Ethical review and approval was not required for the study on human participants in accordance with the local legislation and institutional requirements. The patients/participants provided their written informed consent to participate in this study. Written informed consent was obtained from the individual(s) for the publication of any potentially identifiable images or data included in this article.

## Author contributions

JH advocated the management approach, directed the diagnostic procedures, and wrote the first draft of the manuscript. SK critically evaluated all aspects of the case and contributed to the final draft of the manuscript. All authors contributed to the article and approved the submitted version.

## Conflict of interest

The authors declare that the research was conducted in the absence of any commercial or financial relationships that could be construed as a potential conflict of interest.

## Publisher’s note

All claims expressed in this article are solely those of the authors and do not necessarily represent those of their affiliated organizations, or those of the publisher, the editors and the reviewers. Any product that may be evaluated in this article, or claim that may be made by its manufacturer, is not guaranteed or endorsed by the publisher.

## Supplementary material

The Supplementary material for this article can be found online at: https://www.frontiersin.org/articles/10.3389/fneur.2023.1179596/full#supplementary-material

Supplementary Video S1 Interview just prior to jugular venoplasty demonstrating severe tic disorder.

Supplementary video S2 Interview one week post jugular venoplasty catching short lived clinical improvement.

Supplementary video S3 Interview showing sustained benefit 4.5 years following surgery to decompress the left jugular vein.
